# Biochemical, Pharmacological, and Structural Characterization of New Basic **P** **L** **A**_2_ Bbil-TX from *Bothriopsis bilineata* Snake Venom

**DOI:** 10.1155/2013/612649

**Published:** 2012-12-30

**Authors:** Victor Corasolla Carregari, Rafael Stuani Floriano, Lea Rodrigues-Simioni, Flavia V. Winck, Paulo Aparecido Baldasso, Luis Alberto Ponce-Soto, Sergio Marangoni

**Affiliations:** ^1^Department of Biochemistry, Institute of Biology (IB), Faculty of Medical Sciences, State University of Campinas (UNICAMP), Campinas, SP, Brazil; ^2^Department of Pharmacology, Faculty of Medical Sciences, State University of Campinas (UNICAMP), Campinas, SP, Brazil; ^3^Max Planck Institute of Molecular Plant Physiology and University of Potsdam, Potsdam, Germany

## Abstract

Bbil-TX, a PLA_2_, was purified from *Bothriopsis bilineata* snake venom after only one chromatographic step using RP-HPLC on **μ**-Bondapak C-18 column. A molecular mass of 14243.8 Da was confirmed by Q-Tof Ultima API ESI/MS (TOF MS mode) mass spectrometry. The partial protein sequence obtained was then submitted to BLASTp, with the search restricted to PLA_2_ from snakes and shows high identity values when compared to other PLA_2_s. PLA_2_ activity was presented in the presence of a synthetic substrate and showed a minimum sigmoidal behavior, reaching its maximal activity at pH 8.0 and 25–37°C. Maximum PLA_2_ activity required Ca^2+^ and in the presence of Cd^2+^, Zn^2+^, Mn^2+^, and Mg^2+^ it was reduced in the presence or absence of Ca^2+^. Crotapotin from *Crotalus durissus cascavella* rattlesnake venom and antihemorrhagic factor DA2-II from *Didelphis albiventris* opossum sera under optimal conditions significantly inhibit the enzymatic activity. Bbil-TX induces myonecrosis in mice. The fraction does not show a significant cytotoxic activity in myotubes and myoblasts (C2C12). The inflammatory events induced in the serum of mice by Bbil-TX isolated from *Bothriopsis bilineata* snake venom were investigated. An increase in vascular permeability and in the levels of TNF-a, IL-6, and IL-1 was was induced. Since Bbil-TX exerts a stronger proinflammatory effect, the phospholipid hydrolysis may be relevant for these phenomena.

## 1. Introduction

Viperidae snakes are represented in South America by *Crotalus, Bothrops, Bothriopsis *and* Lachesis*. *Bothriopsis bilineata* is the endemic and rare bothropic snake species [1].

The envenomation is characterized by a generalized inflammatory state. The normal reaction to envenomation involves a series of complex immunologic cascades that ensures a prompt protective response to venom in humans [2]. Although activation of the immune system during envenomation is generally protective, septic shock develops in a number of patients as a consequence of excessive or poorly regulated immune response to the injured organism [3]. This imbalanced reaction may harm the host through a maladaptitive release of endogenous mediators that include cytokines and nitric oxide.

PLA_2_s are abundant in snake venoms and have been widely employed as pharmacological tools to investigate their role in diverse pathophysiological processes. Viperid and crotalid venoms contain PLA_2_s with the ability to cause rapid necrosis of skeletal muscle fibers, thus being referred to as myotoxic PLA_2_s [4]. Local inflammation is a prominent characteristic of snakebite envenomations by viperid and crotalid species [5].

Furthermore, PLA_2_ myotoxins are relevant tools for the study of key general inflammatory mechanisms. High levels of secretory PLA_2_ (sPLA_2_) are detected in a number of inflammatory disorders in humans, such as bronchial asthma [6], allergic rhinitis [7], septic shock [8], acute pancreatitis [9], extensive burning [10], and autoimmune diseases [11]. In addition, increased expression and release of sPLA_2_ have been found in rheumatoid arthritis, inflammatory bowel diseases, and atherosclerosis [12, 13]. Mechanisms involved in the proinflammatory action of sPLA_2_ are being actively investigated, and most of this knowledge is based on studies using purified venom PLA_2_s.

This paper describes the isolation and biochemical and pharmacological characterization of new PLA_2_s from *Bothriopsis bilineata* venom, Bbil-TX, and also the study of its various toxic activities, including myotoxicity, cytotoxicity, and inflammation.

## 2. Materials and Methods

### 2.1. Venom and Reagents

*Bothriopsis bilineata* venom was donated by Dr. Corina Vera Gonzáles. All chemicals and reagents used in this work were of analytical or sequencing grade and purchased from Sigma-Aldrich Co. (St. Louis, MO, USA).

### 2.2. Reversed-Phase HPLC (RP-HPLC)

Five milligrams of the whole venom from *Bothriopsis bilineata* was dissolved in 200 *μ*L ammonium bicarbonate 0.2 M pH 8.0. The resulting solution was clarified by centrifugation and the supernatant was applied to a *μ*-Bondapak C18 column (0.78 × 30 cm; Waters 991—PDA system). Fractions were eluted using a linear gradient (0–100%, v/v) of acetonitrile (solvent B) at a constant flow rate of 1.0 mL/min over 40 min. The elution profile was monitored at 280 nm, and the collected fractions were lyophilized and conserved at −20°C.

### 2.3. PLA_2_ Activity

PLA_2_ activity was measured using the assay described by Holzer and Mackessy, [14] modified for 96-well plates. The standard assay mixture contained 200 *μ*L of buffer (10 mM tris-HCl, 10 mM CaCl_2_ and 100 mM NaCl, pH 8.0), 20 *μ*L of substrate (4-nitro-3-octanoyloxy-benzoic acid), 20 *μ*L of water, and 20 *μ*L of Bbil-TX in a final volume of 260 *μ*L. After adding Bbil-TX (20 *μ*g), the mixture was incubated for up to 40 min at 37°C, with the reading of absorbance at intervals of 10 min. The enzyme activity, expressed as the initial velocity of the reaction (*V*_*o*_), was calculated based on the increase of absorbance after 20 min. The optimum pH and temperature of the PLA_2_ were determined by incubating the enzyme in four buffers of different pH values (4–10) and at different temperatures, respectively. The effect of substrate concentration (0.1, 0.2, 0.3, 0.5, 1, 2, 5, 10, 20, and 30 mM) on enzyme activity was determined by measuring the increase of absorbance after 20 min. The inhibition of PLA_2_ activity by crotapotins from *Crotalus durissus cascavella *and DAII-2 from *Didephis albiventris* serum was determined by preincubating the protein (Bbil-TX) and each inhibitor for 30 min at 37°C prior to assaying the residual enzyme activity under optimal conditions. All assays were done in triplicate and the absorbances at 425 nm were measured with a VersaMax 190 multiwell plate reader (Molecular Devices, Sunnyvale, CA, USA).

### 2.4. Electrophoresis

Tricine SDS-PAGE in a discontinuous gel and buffer system was used to estimate the molecular mass of the proteins, under reducing and nonreducing conditions [15].

### 2.5. Amino Acid Analysis

Amino acid analysis was performed on a Pico-Tag Analyzer (Waters Systems) as described by [16]. The purified Bbil-TX sample (30 *μ*g) was hydrolyzed at 105°C for 24 h in 6 M HCl (Pierce sequencing grade) containing 1% phenol (w/v). The hydrolyzates were reacted with 20 *μ*L of derivatization solution (ethanol : triethylamine : water : phenylisothiocyanate, 7 : 1 : 1 : 1, v/v) for 1 h at room temperature, after which the PTC-amino acids were identified and quantified by HPLC, by comparing their retention times and peak areas with those from a standard amino acid mixture.

### 2.6. Determination of the Molecular Mass of the Purified Protein by Mass Spectrometry

An aliquot (4.5 *μ*L) of the protein was inject by C18 (100 *μ*m × 100 mm) RP-UPLC (nanoAcquity UPLC, Waters) coupled with nanoelectrospray tandem mass spectrometry on a Q-T of Ultima API mass spectrometer (MicroMass/Waters) at a flow rate of 600 nl/min. The gradient was 0–50% acetonitrile in 0.1% formic acid over 45 min. The instrument was operated in MS continuum mode and the data acquisition was from *m*/*z* 100–3,000 at a scan rate of 1 s and an interscan delay of 0.1 s. The spectra were accumulated over about 300 scans and the multiple charged data produced by the mass spectrometer on the *m*/*z* scale were converted to the mass (molecular weight) scale using maximum-entropy-based software (1) supplied with Masslynx 4.1 software package. The processing parameters were: output mass range 6,000–20,000 Da at a “resolution” of 0.1 Da/channel; the simulated isotope pattern model was used with the spectrum blur width parameter set to 0.2 Da and the minimum intensity ratios between successive peaks were 20% (left and right). The deconvoluted spectrum was then smoothed (2 × 3 channels, Savitzky Golay smooth) and the mass centroid values obtained using 80% of the peak top and a minimum peak width at half height of 4 channels.

### 2.7. Analysis of Tryptic Digests

The protein was reduced (DTT 5 mM for 25 min to 56°C) and alkylated (Iodoacetamide 14 mM for 30 min) prior to the addition of trypsin (Promega's sequencing grade modified). After trypsin addition (20 ng/*μ*L in ambic 0.05 M), the sample was incubated for 16 hr at 37°C. To stop the reaction, formic acid 0.4% was added and the sample centrifuged at 2500 rpm for 10 min. The pellet was discarded and the supernatant dried in a speed vac. The resulting peptides were separated by C18 (100 *μ*m × 100 mm) RP-UPLC (nanoAcquity UPLC, Waters) coupled with nanoelectrospray tandem mass spectrometry on a Q-Tof Ultima API mass spectrometer (MicroMass/Waters) at a flow rate of 600 nl/min. Before performing a tandem mass spectrum, an ESI/MS mass spectrum (TOF MS mode) was acquired for each HPLC fraction over the mass range of 100–2000 *m*/*z*, in order to select the ion of interest; subsequently, these ions were fragmented in the collision cell (TOF MS/MS mode).

Raw data files from LC-MS/MS runs were processed using Masslynx 4.1 software package (Waters) and analyzed using the MASCOT search engine version 2.3 (Matrix Science Ltd.) against the snakes database, using the following parameters: peptide mass tolerance of ±0.1 Da, fragment mass tolerance of ±0.1 Da, and oxidation as variable modifications in methionine and trypsin as enzyme.

### 2.8. Myotoxic Activity

Groups of four Swiss mice (18–20 g) received an intramuscular (i.m.) or an intravenous (i.v.) injection of variable amounts of the Bbil-TX. Samples (50 *μ*L) containing 0.1, 1, and 5 *μ*g of the PLA_2_ Bbil-TX were injected in the right gastrocnemius. A control group received 50 *μ*L of PBS. At different intervals, blood was collected from the tail into heparinized capillary tubes after 2, 4, 6, 9, 12 and 24 hours, and the plasma creatine kinase (CK; EC 2.7.3.2) activity was determined by a kinetic assay Ck-Nac, (creatine kinase, Beacon, Diagnostics, Germany). To the reaction mixture 10 *μ*L of the plasma obtained by centrifugation from mice blood was added. The solution is incubated for 2 minutes and reads at 430 nm. The results were expressed as U/L according to the manufacturer.

### 2.9. Cytotoxicity Assays

Cytotoxic activity was assayed on murine skeletal muscle C2C12 myoblasts and myotubes (ATCC CRL-1772). Variable amounts of Bbil-TX were diluted in assay medium (Dulbecco's Modified Eagle's Medium supplemented with 1% fetal-calf serum) and added to cells in 96-well plates, in 150 *μ*L. Controls for 0 and 100% toxicity consisted of assay medium, and 0.1% Triton X-100, respectively. After 3 h at 37°C, a supernatant aliquot was collected for determination of lactic dehydrogenase (LDH; EC 1.1.1.27) activity released from damaged cells, using a kinetic assay (Wiener LDH-P UV). Experiments were carried out in triplicate.

### 2.10. Edema-Forming Activity

The ability of Bbil-TX to induce edema was studied in groups of five Swiss mice (18–20 g). Fifty microliters of phosphate-buffered saline (PBS; 0.12 M NaCl, 0.04 M sodium phosphate, pH 7.2) with Bbil-TX (0.1; 1 and 5 *μ*g/paw) were injected in the subplantar region of the right footpad. The control group received an equal volume of PBS alone. The paw swelling was measured with an Electronic Caliper Series 1101 (INSIZE LTDA, SP, Brazil) at 0.5, 1, 3, 6, 9, and 24 h after administration. Edema was expressed as the percentage increase in the size of the treated group to that of the control group at each time equal to 24 hrs.

### 2.11. Cytokines

The levels of cytokines IL-6 and IL-1 in the serum from BALB/c mice were assayed by a two-site sandwich enzyme-like immunosorbent assay (ELISA) as described by [17]. In brief, ELISA plates were coated with 100 *μ*L (1 *μ*g/mL) of the monoclonal antibodies anti-IL-6 and anti-IL-1 placed in 0.1 M sodium carbonate buffer (pH 8.2), and incubated for 6 hours at room temperature. The wells were then washed with 0.1% phosphate-buffered saline (PBS/Tween 20) and blocked with 100 *μ*L of 10% fetal-calf serum (FCS) in PBS for 2 hours at room temperature. After washing, duplicate sera samples of 50 *μ*L were added to each well. After 18 hours of incubation at 4°C, the wells were washed and incubated with 100 *μ*L (2 *μ*g/mL) of the biotinylated monoclonal antibody anti-IL-6 and anti-IL-1 as a second antibody for 45 minutes at room temperature. After a final wash, the reaction was developed by the addition of orthophenyldiamine (OPD) to each well. Optical densities were measured at 405 nm in a microplate reader. The cytokine content of each sample was read from a standard curve established with the appropriate recombinant cytokines (expressed in picograms per millilitre). The minimum levels of each cytokine detectable in the conditions of the assays were 10 pg/mL for IL-6 and IL-1.

To measure the cytotoxicity of TNF-*α* present in the serum from BALB/c mice, a standard assay with L-929 cells, a fibroblast continuous cell line, was used as described previously by [18]. The percentage cytotoxicity was calculated as follows: (A_control_ − A_sample_/A_control_) × 100. Titres were calculated as the reciprocal of the dilution of the sample in which 50% of the cells in the monolayer were lysed. TNF-*α* activity is expressed as units/mL, estimated from the ratio of a 50% cytotoxic dose of the test to that of the standard mouse recombinant TNF-*α*.

### 2.12. Statistical Analysis

Results are reported as means ± SEM. The significance of differences between the means was assessed by ANOVA followed by Dunnett's test when various experimental groups were compared with the control group. A value of *P* < 0.05 indicated significance.

## 3. Results

**Figure 1:**
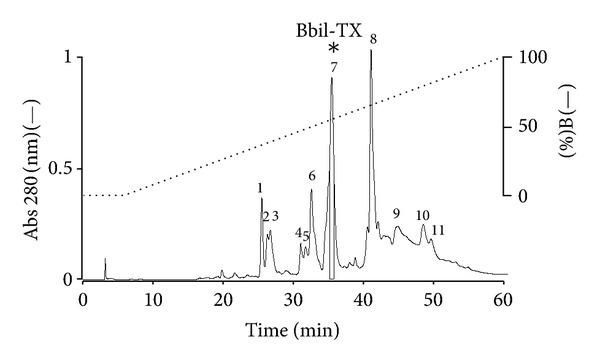
Elution profile of *Bothriopsis bilineata* venom by RP-HPLC on a *μ*-Bondapack C18 column. Fraction 7 (Bbil-TX) contained PLA_2_ activity.

Fractionation of *Bothriopsis bilineata* venom by RP-HPLC on a *μ*-Bondapak C18 column resulted in eleven peaks (1–11) (Figure 1). The 11 peaks were screened for myotoxic and PLA_2_ activities. Peak 7 caused local myotoxicity at concentrations ranging from 0.1 to 5 *μ*g/mL in mouse gastrocnemius muscle. In addition, peak 7, named Bbil-TX-I (*Bothriopsis bilineata* toxin) showed high PLA_2_ activity and was selected for biochemical and pharmacological characterization. The purity of this peak was confirmed by rechromatography on an analytical RP-HPLC *μ*-Bondapack C18 column, showing the presence of only one peak and by Tricine SDS-PAGE, which revealed the presence of one electrophoretic band with Mr around 15 kDa, in the absence and presence of DTT (1 M) (data not shown).

**Figure 2:**
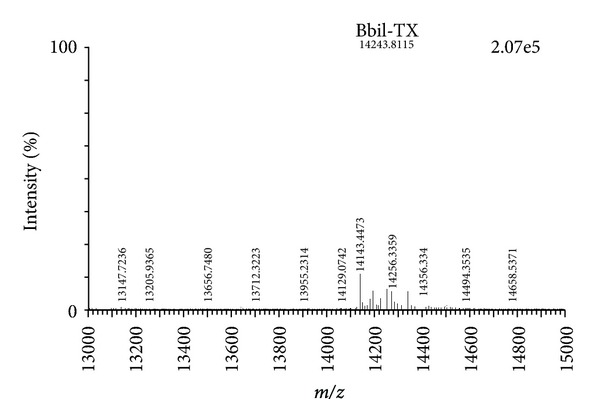
Mass determinations of Bbil-TX by mass spectrometry, using a Q-Tof Ultima API ESI/MS (TOF MS mode).

Q-Tof Ultima API ESI/MS (TOF MS mode) mass spectrometry analysis confirmed the homogeneity of the peak Bbil-TX and determined the exact molecular mass of 14243.8 Da (Figure 2). This value of molecular mass was used in calculating the molar concentrations of toxin used in the experiments described below.

The alkylated and reduced protein was digested with trypsin and the resulting tryptic peptides (10) were fractionated by RP-UPLC (nanoAcquity UPLC, Waters) (data not shown). Before performing a tandem mass spectrum, an ESI/MS mass spectrum (TOF MS mode) was acquired for each HPLC fraction over the mass range of 100–2000 *m*/*z*, in order to select the ion of interest; subsequently, these ions were fragmented in the collision cell (TOF MS/MS mode). The data files obtained from LC-MS/MS runs were processed using the Masslynx 4.1 software package (Waters) and analyzed using the MASCOT search engine version 2.3 (http://www.matrixscience.com/).  Table 1 shows the deduced sequence and measured masses of alkylated peptides obtained for Bbil-TX PLA_2_. Isoleucine and leucine residues were not discriminated in any of the sequences reported since they were indistinguishable in low energy CID spectra. Because of the external calibration applied to all spectra, it was also not possible to resolve the 0.041 Da difference between glutamine and lysine residues, except for the lysine that was deduced based on the cleavage and missed cleavage of the enzyme.

The ten peptides obtained in Q-Tof Ultima API ESI/MS (TOF MS mode) mass spectrometry of the Bbil-TX PLA_2_ were submitted to the NCBI database, using the protein search program BLASTp with the search being restricted to the sequenced proteins from the basic protein with phospholipase A_2_ activity family. Based on the positional matches of the de novo sequenced peptides with other homologous proteins, it was possible to deduce the original positions of these peptides in the native protein (Figure 4).

**Figure 3:**
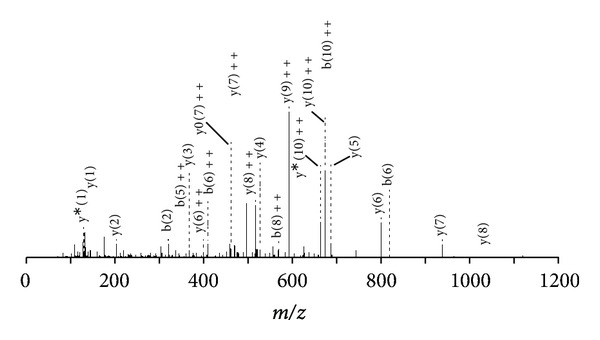
MS/MS spectrum of the doubly charged tryptic ion of *m*/*z* 1504.5356. Ion of the major sequence-specific *y*-ion series and of a minor series of the complementing *b*-ions CCFVHDCCYGK, from which the sequence of Bbil-TX tag was deduced.

The results of the primary structures show that Bbil-TX PLA_2_ is composed of 122 amino acid residues and shares the conserved sequence domains common to PLA_2_ group, including the 14 cysteines, the calcium-binding site located on (Y)27, (G)28, (C)31, and (G)32, and the catalytic network commonly formed by (H)48, (D)49, (Y)52, and (D)90. A comparative analysis of the sequence of Bbil-TX PLA_2_ with other neurotoxins “*ex vivo*” and myotoxic PLA_2_s belonging to Viperidae family, LmTX-I and LmTX-II (*Lachesis muta muta*) [19] and *Crotalus scutulatus scutulatus* (Mojave rattlesnake) (accession number P62023), showed similarity of 81.1–91.0%. (SwissProt database http://br.expasy.org/). The tandem mass spectra shown in Figure 3, relative to the peptide eluted in fraction 3 having the sequence C C F V H D C C Y G K, allows to classify both enzymes as PLA_2_.

Amino acid analysis revealed the following composition of Bbil-TX PLA_2_: D/11, T/7, S/2, E/12, P/9, G/10, A/7, C/14, V/4, M/2, I/3, L/7, Y/9, F/4, K/12, H/1, and R/6. The PLA_2_ activity was examined in the *Bothopsis bilineata* venom and in BbilTX-I using the synthetic substrate 4-nitro-3(octanoyloxy) benzoic acid [14, 19]. The PLA_2_ activity was higher in Bbil-TX (24.75 ± 2.68 nmols/min/mg) when compared with the whole venom (8.15 ± 1.24 nmols/min/mg) (Figure 5(a)). Under the conditions used, Bbil-TX showed a discrete sigmoidal behavior (Figure 5(f)), mainly at low substrate concentrations. Maximum enzyme activity occurred at 35–40°C (Figure 5(c)) and the pH optimum was 8.0 (Figure 5(d)). PLA_2_s require Ca^+2^ for full activity, with only 1 mM of Ca^+2^ needed for Bbil-TX to present phospholipase activity. The addition of Mg^2+^, Cd^2+^, and Mn^2+^ (10 mM) in the presence of low Ca^2+^ concentration (1 mM) decreases the enzyme activity. The substitution of Ca^2+^ by Mg^2+^, Cd^2+^, and Mn^2+^ also reduced the activity to levels similar to those in the absence of Ca^2+^ (Figure 5(e)).

The crotapotins are pharmacologically inactive and nonenzymatic acid protein, binds specifically of the PLA_2_ inhibited the activity. An isoform of *Crotalus durissus cascavella *F3 and antihaemorragic factor DA2-II from *Didelphis albiventris,* significantly inhibit the Bbil-TX PLA_2_ activity (Figure 5(b)).

The local myotoxic effect (i.m.) *in vivo* was observed with PLA_2_ Bbil-TX studied. It was observed that the PLA_2_ induced a conspicuous effect evidenced by the rapid elevation of plasma CK activity through a time course, reaching its maximum effect 2 h after injection and returning to normal levels after 24 h (Figure 6(a)). Our results showed that the PLA_2 _Bbil-TX did not show systemic myotoxic effect (i.v.) (Figure 6(b)).

In a concentration of 5, 10, 20, and 40 *μ*g/well (150 *μ*L), the PLA_2_ Bbil-TX showed low cytotoxicity in skeletal muscle myoblasts and myotubes (25.49 ± 2.3% and 29.05 ± 3.45%, resp.) in a concentration of 40 mg/well (150 *μ*L) (Figure 6(c)).

Compared to PBS-injected animals, those which received subplantar injections of the Bbil-TX (0.1, 1, and 5 *μ*g/paw) presented marked paw edema (Figure 7(a)). Maximal activity was attained 1 h to the Bbil-TX after injection and receded to normal levels after 24 h. The level of edema induction by 5 *μ*g of PLA_2_ 1 hour after administration was 61.57%, showing a dose-dependent activity. To further analyze the mechanisms of the inflammatory events induced by Bbil-TX (0.1 *μ*g), TNF-*α*, IL-6, and IL-1 concentrations were measured in the serum. TNF-*α* levels were increased 1 h after injection of Bil-TX and no detectable production was observed at the later time intervals studied (Figures 7(b), 7(c), and 7(d)). Bbil-TX caused a significant increase in IL-6 release between 1 and 3 h, respectively, in serum collected after injection of venom compared with the control (Figure 7(c)). However, increased levels of IL-1 were detected between 1, 3, 6, and 12 h, respectively (Figure 7(d)).

## 4. Discussion

The purification procedure for basic PLA_2_s developed by [20–22] showed to be also efficient for the obtainment of neurtoxin “*ex vivo*” and myotoxin from *Bothriopsis bilineata* snake venom. Fractionation of this crude venom by single-step chromatography in a column *μ*-Bondapack C-18 coupled to a system of reversed-phase HPLC was carried out and as a result of the proposed method, several toxins have been efficiently purified. Fraction 7 was named Bbil-TX (PLA_2_). SDS-PAGE showed evidence that Bbil-TX isolated PLA_2_s have an Mr of ~14 kDa for the monomers, similar to basic PLA_2_s isolated from other venoms (data not shown) [23]. The conserved residues Y28, G30, G32, D49, H48, and Y52 are directly or indirectly linked in the catalyses of the Bbil-TX.

The molecular masses obtained by mass spectrometry showed to be similar to that of other snake venom PLA_2_s [22, 24, 25]. The amino acid composition of the Bbil-TX PLA_2_ toxin suggests the presence of 14 half-Cys residues, providing the basis for a common structural feature of PLA_2_ in the formation of its seven disulfide bridges [20, 21, 26] and a high content of basic and hydrophobic residues, that provides a explication important in the interaction of the PLA_2_ with negatively charged phospholipids of cells membranes [27]. Such an interaction is important to explain the effect of these enzymes on different cells types, both prokariotes and eukariotes [28, 29].

**Figure 4:**
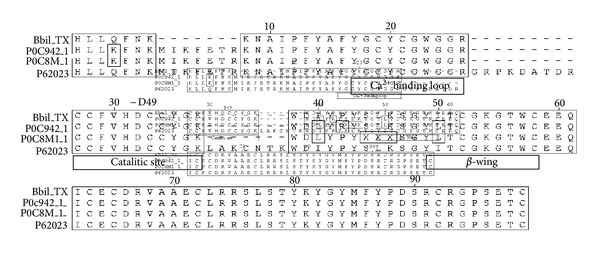
Alignment of the deduced amino acid sequence of the new PLA_2_ Bbil-TX with PLA_2_ presents in venom of *Lachesis muta muta* (accession number P0C8 M_1 and P0C942_1) [19] and *Crotalus scutulatus scutulatus* (Mojave rattlesnake) (accession number P62023). Nondetermined amino acid residues are indicated by (X); boxed amino acid residues are identical. The highlighted amino acid residues belong to PLA_2_ conserved domain Ca^2+^-binding loop, the catalytic site, and the region *β*-wing.

**Figure 5:**
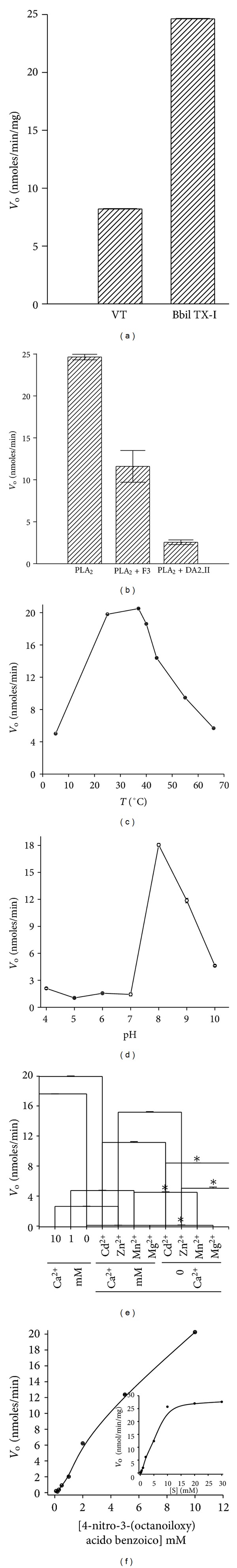
(a) PLA_2_ activity of *Bothriopsis bilineata* venom and peak 7 (Bbil-TX); (b) the inhibitory effect of the antihemorrhagic factor DAII-2 and the crotapotin F3 on PLA_2_ activity Bbil-TX; (c) effect of temperature on the PLA_2_ activity of Bbil-TX; (d) effect of pH on Bbil-TX activity; (e) influence of ions (10 mM each) on PLA_2_ activity in the absence or presence of 1 mM Ca^2+^; (f) effect of substrate concentration on the kinetics of BbilTX (PLA_2_) activity. The inset shows the curve shape at low substrate concentrations. The results of all experiments are the mean ± SE, of three determinations (*P* < 0.05).

Comparison of the amino acid sequence of Bbil-TX PLA_2_ showed high homology with other neurotoxic and myotoxic PLA_2_s from *Lachesis* and *Crotalus* genera (Figure 4). Sequence homology studies had shown that there are extremely conserved positions in the PLA_2_s. In positions 1 and 2, there is a predominance of the amino acid sequence (HL), in position 4 (Q), and in positions 5 to 7 (FNK). One of the highly conserved regions in the amino acid sequences of PLA_2_ is the Ca^2+^-binding loop, segment from …YGCYCGXGG… and HD(49)CC. The calcium ion is coordinated by three main chain oxygen atoms from residues (Y)28, (G)30, (G)32, and two carboxylate oxygen atoms of (D)49. Two generally conserved solvent water molecules complete the coordination sphere of the calcium ion forming a pentagonal bipyramidal geometry. It is believed that two disulfide bridges (C)27–(C)119 and (C)29–(C)45 ensure the correct relative orientation of the calcium-binding loop in relation to the amino acids of the catalytic network [30]. The residues (H)48, (Y)52, and (D)99 which are responsible for catalytic activity have an ideal stereochemistry with the presence of the so-called “catalytic network”, a system of hydrogen bonds which involves the catalytic triad [30, 31]. Residues forming the Ca^2+^-binding loop and the catalytic network of Bbil-TX PLA_2_ show a high conservation grade, reflecting the nondecreased catalytic activity.

The PLA_2_ activity was shown to be higher in Bbil-TX PLA_2_ (24.75 ± 2.28 nmoles/min/mg) when compared with the whole venom (8.15 ± 1.24 nmoles/min/mg). PLA_2_ enzyme from snake venom shows classic Michaelis–Menten behavior against micellar substrates [32]. With a synthetic substrate, Bbil-TX PLA_2_ behaved allosterically, especially at low concentrations, which is in agreement with the results obtained by [23] for the PLA_2_ of *Bothrops jararacussu* venom and Damico et al. [19] for the PLA_2_ isoform purified from *Lachesis muta muta *venom. Using the same synthetic nonmicellar substrate, it was also possible to observe that the dependence of activity on substrate concentration was markedly sigmoidal for the PrTX-III from *Bothrops pirajai* [33].

PLA_2_s from crotalic venoms have showed a similar behavior to the one presented by bothropic PLA_2_s with the same substrate used in the kinetic studies to Bbil-TX PLA_2_ [14, 34]. Despite the structural and functional differences among bothropic and crotalic PLA_2_s, both show allosteric behavior in the presence of the same substrate.

The PLA_2_ activity could be verified with different pH levels; the optimum pH of basic PLA_2_s is around 7.0 and 8.5 [32, 35]. Bbil-TX PLA_2_ can be considered basic since its highest activity is evidenced at pH 8.0. Temperature is another kinetic parameter utilized to characterize the PLA_2_ (Asp49). It has been shown that PLA_2_ from *Naja naja naja* is very stable in extreme temperatures such as 100°C [35]. The optimum temperature of Bbil-TX PLA_2_ was around 37°C, but at 40–45°C, the Bbil-TX PLA_2_ activity did not present a huge decrease.

A strict requirement for Ca^2+^ is characteristic of some PLA_2_ [5]. Bbil-TX PLA_2_ showed typical Ca^2+^-dependent PLA_2_ activity similar to other PLA_2_s, and this activity was lower in the presence of other cations. [14, 35–38] observed the same for other PLA_2_ from snake venom.

The crotapotin isoform from *Crotalus durrissus cascavella *(F3) venom inhibit significantly the PLA_2_ activity of Bbil-TX by approximately 50%. Our results are in agreement with the finding by [14, 21, 39] who reported that highly purified crotapotin can inhibit pancreatic, bee, and other snake venom PLA_2_s, and Bonfim et al. [23], who reported that crotapotins from *Crotalus durissus terrificus* (F7), *Crotalus durissus collilineatus* (F3 and F4), and *Crotalus durissus cascavella* (F3 and F4) decreased the catalytic activity of BJ IV (PLA_2_ from *Bothrops jararacussu*) by 50%. Together, these results suggest that crotapotin may bind to bothropic PLA_2_ in a manner similar to that from crotalic PLA_2_.

**Figure 6:**
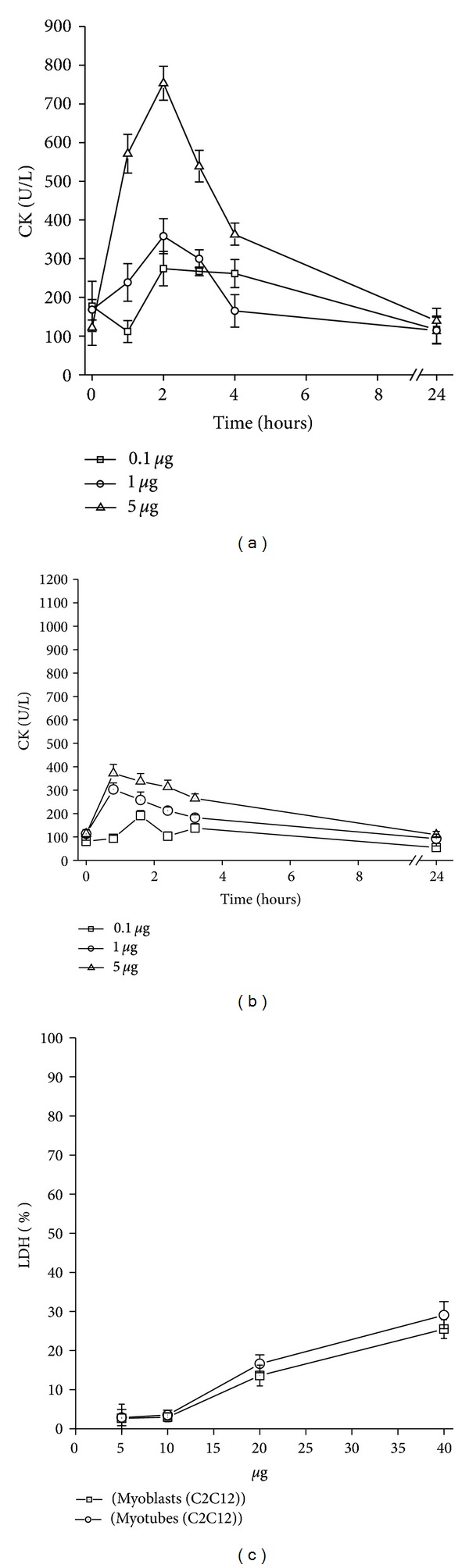
(a, b) Time course of the increments in plasma CK activity after intramuscular (i.m.) or intravenous (i.v.) injection of 0.1, 1, and 5 *μ*g *Bothriopsis bilineata* Bbil-TX; (c) *in vitro* cytotoxic activity of Bbil-TX on murine C2C12 skeletal-muscle myoblasts and myotubes. Cell lysis was estimated by the release of lactic dehydrogenase (LDH) to supernatants, after 3 h of exposure to the toxins. Each point represents mean ± SD of triplicate cell cultures.

**Figure 7:**
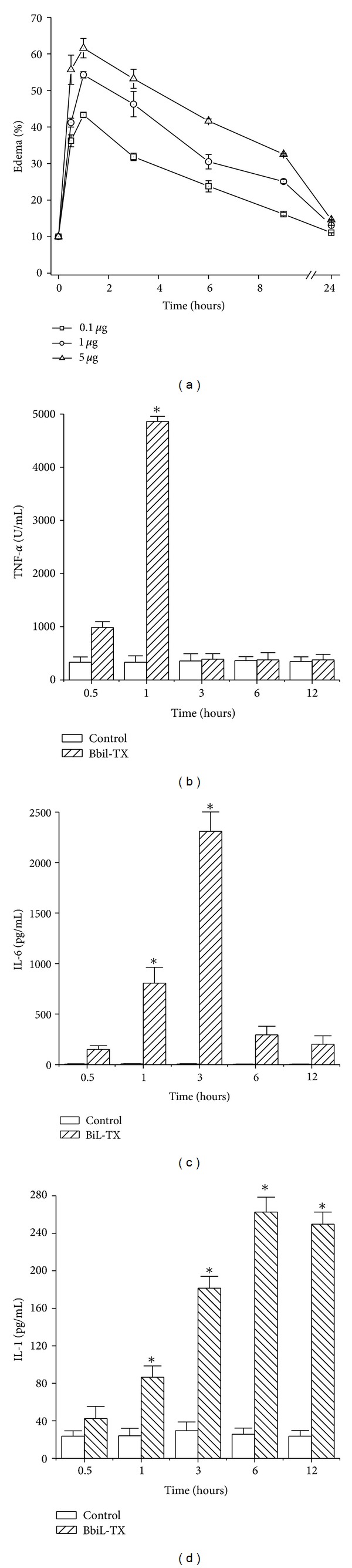
(a) Edema-forming activity of Bbil-TX in mice. Induction of edema by toxins (0.1, 1, and 5 *μ*g/mL), injected s.c. in the footpad of mice. At various time intervals the increase in footpad volume, as compared to controls, was expressed as percent edema. Each point represents the mean ± SD of four animals. Levels of TNF-*α* (b), IL-6 (c), and IL-1 (d), in the serum after injection of Bbil-TX. Animals were injected i.m. with Bbil-TX (1.0 mg/kg) or sterile saline alone (control) in a final volume of 100 *μ*L. IL-1 and IL-6 were quantified by specific ELISA, and TNF-*α* was quantified by cytotoxic activity on L929 cells in serum collected at the indicated time intervals after Bbil-TX or saline injection as described in Materials and Methods. Each bar represents mean ± SEM of 7 animals. *P < 0.05 when compared with the corresponding control groups.

Bbil-TX PLA_2_ increases the plasmatic CK levels after i.m. injection (Figure 6(a)), revealing drastic local myotoxicity. This myotoxicity induced by snake venoms, including *Botrhiopsis bilineata*, may result from the direct action of myotoxins on the plasma membranes of muscle cells, or indirectly, as a consequence of vessel degenerations and ischemia caused by hemorrhagins or metaloproteases. Bbil-X PLA_2_ contributes significantly to local myotoxic action *in vivo*. It was already demonstrated that the snake venom PLA_2_s are the principal cause of local damage [40]. Myotoxic PLA_2_s affect directly the plasma membrane integrity of muscle cells, originating an influx of Ca^2+^ ions to the citosol that starts several degenerative events with irreversible cell injures [41]. The binding sites of myotoxins on the plasma membranes are not clearly established, although two types have been proposed: (a) negatively charged phospholipids [42], present on membranes of several cell types, explaining the high *in vitro* cytotoxic action of these enzymes [28, 43, 44], and (b) protein receptors, which make muscle cells more susceptible to myotoxin action [28].

All these biological effects induced by the toxin occur in the presence of a measurable PLA_2_ activity. Although the catalytic activity of PLA_2_s contributes to pharmacological effects, it is not a prerequisite [21, 26, 29]. However, further studies are necessary to identify the structural determinants involved in these pharmacological activities.

Some authors, [21, 41, 45, 46], have proposed several models to explain PLA_2_ catalytic and pharmacological activities. In these models PLA_2_ has two separated places; one is responsible for catalytic activity and the other for biological activity expression. According to them, the pharmacological place would be located on the surface of PLA_2_ molecules. According to the model proposed by [47], the anti-coagulant place would be located in a region between the 53 and 76 residues, considering this region charged positively in the PLA_2_ with high anticoagulant activity. In PLA_2_ with moderate or low anticoagulant activity, there is a predominancy of negative charges.

Further research in identifying target proteins will help determine details of the mechanisms of the pharmacological effects at the cellular and molecular levels [48]. Studies in these areas will result in new, exciting, and innovative opportunities and avenues in the future, both in finding answers to the toxicity of PLA_2_ enzymes and in developing proteins with novel functions.

PLA_2_s from snake venoms exert a large number of pharmacological activities due to a process of accelerated evolution through which a high mutational rate in the coding regions of their genes has allowed the development of new functions, mainly associated with the exposed regions of the molecules [29]. The integral analysis of the inflammation elicited by Bbil-TX in the mouse serum performed in the present study allowed a parallel evaluation of the increase in microvascular permeability and the production of various inflammatory mediators. Bbil-TX induced an increase in vascular permeability in the paw of mice. This is in agreement with previous observations on the edema-forming activity of similar molecules in the rodent footpad model [49, 50].

The increase of vascular permeability was detected early after Bbil-TX injection and developed rapidly, indicating that the observed plasma extravasation is primarily due to formation of endothelial gaps in vessels of microcirculation. The main edema formation occurred 1 h after the injection of Bbil-TX with constant decrease. Bbil-TX caused paw edema in mice with a time course similar to that reported for other PLA_2_s from* Bothrops* venoms in mice and rats, that is, a fairly rapid onset (generally ≤ 3 h to peak) followed by a gradual decline over the following 24 h [51–54].

The mediators involved in this effect of Bbil-TX myotoxin were not addressed in this study. However, the immediate plasma extravasation in response to Bbil-TX strongly suggests the involvement of vasoactive mediators derived from mast-cell granules. This strongly suggests that enzymatic phospholipid hydrolysis plays a significant role in this event.

Cytokines, such as IL-1, IL-6, and TNF-*α*, are also relevant mediators for leukocyte migration and participate in several inflammatory conditions. Our results showed that Bbil-TX induced an increase in TNF-*α*, IL-6, and IL-1 in the serum [55]. Thus, our results suggest that IL-1 may contribute to the leukocyte influx induced by Bbil-TX. In addition, the similarity observed in the time course of IL-6 and IL-1 increase in the serum may indicate a positive regulatory role for IL-1 on the release of IL-6 induced by Bbil-TX. IL-6, an important mediator of inflammation, causes leukocytosis characterized by a rapid neutrophilia by releasing of PMN leukocytes from the bone marrow [56, 57]. In addition, IL-6 upregulates intercellular-adhesion-molecule-1 (ICAM-1) expression by endothelial cells but decreases the levels of L-selectin on circulating PMN leukocytes contributing to firm adhesion, the next step of cell migration [58].

TNF-*α* is also likely to be involved in leukocyte infiltration induced by Bbil-TX, since the PLA_2_ caused a significant increase of TNF-*α* levels in the serum. TNF-*α* is likely to induce the expression of E-selectin, CD11b/CD18, and intercellular adhesion molecule-1 (ICAM-1) and triggers the release of several cytokines such as IL-1 and IL-6 and eicosanoids. Thus, our results suggest that TNF-*α* may have a role in the expression of CD18 and the release of other cytokines following Bbil-TX injection, thereby being relevant for neutrophil influx and for increase of vascular permeability. It is interesting that TNF-*α* and IL-6, as well as IL-1, may induce or potentiate the expression and release of group IIA PLA_2_s [59, 60].

In conclusion, Bbil-TX induces a marked inflammatory reaction in the mouse serum. Since basic myotoxic PLA_2_s are abundant in snake venoms, these toxins must play a relevant role in the proinflammatory activity that characterizes this venom. The fact that Bbil-TX elicited a stronger inflammatory reaction argues in favor of a role of enzymatic phospholipid hydrolysis in this phenomenon, either through the direct release of arachidonic acid from plasma membranes or through activation of intracellular processes in target cells.

Accumulating evidences have strongly shown that venom PLA_2_s are among the major mediators of myonecrosis [40], hemolysis, mast cell degranulation, and edema formation [3].

PLA_2_s isolated from *Bothrops* venoms are frequently myotoxic [26] and can cause edema in rats and mice [39, 45, 49, 54]. These results suggest that, for some PLA_2_s, catalytic activity plays a role in the edematogenic effect.

## Figures and Tables

**Table 1:** Sequence obtained by MS/MS based on the alkylated tryptic peptides derived. The peptides were separated and sequenced by mass spectrometry.

Residue number	Mass (Da) expected	Amino acid sequence	Mass (Da) calculated
1–7	898.5293	HLLQFNK	898.5025
16–33	2157.9352	NAIPFYAFYGCYCGWGGR	2157.9189
43–53	1504.5356	CCFVHDCCYGK	1504.5356
61–69	1183.6216	WDIYPYSLK	1183.5913
70–77	884.4277	SGYITCGK	884.4062
78–90	871.8404	GTWCEEQICECDR	1741.6494
91–98	973.5307	VAAECLRR	973.5127
98–104	853.4780	RSLSTYK	853.4657
105–114	1297.5059	YGYMFYPDSR	1297.5437
115–122	965.3514	CRGPSETC	965.3695
